# Searching for Infectious Foci in Intensive Care Patients: Diagnostic Yield of Computed Tomography and Prognostic Value of Clinical and Laboratory Chemical Parameters

**DOI:** 10.3390/jcm14072180

**Published:** 2025-03-22

**Authors:** Ron Martin, Dieter Fedders, Robert Winzer, Jonas Roos, Alexander Isaak, Julian Luetkens, Daniel Thomas, Daniel Kuetting

**Affiliations:** 1 Department of Plastic and Hand Surgery, Burn Care Center, BG Klinikum Bergmannstrost Halle, Merseburger Str. 165, 06112 Halle, Germany; 2 Department of Radiology and Neuroradiology, Chemnitz Hospital, 09116 Chemnitz, Germany; 3 Department of Nuclear Medicine, University Hospital of Dresden, 01307 Dresden, Germany; 4 Department of Orthopedics and Trauma Surgery, University Hospital of Bonn, 53127 Bonn, Germany; 5 Department of Radiology, University Hospital of Bonn, 53127 Bonn, Germany; 6 Department of Radiology, St.-Vinzenz Hospital Cologne, 50733 Cologne, Germany; daniel.thomas@cellitinnen.de

**Keywords:** computed tomography, infectious foci, intensive care unit, sepsis

## Abstract

**Background/Objectives:** Radiological imaging is crucial in intensive care settings, particularly for the differential diagnosis of fever and sepsis. Computed tomography (CT) is the preferred method for detecting infectious foci in critically ill ICU patients. **Methods:** This study prospectively analyzed non-ECG-gated chest and abdominal CT scans from ICU patients to assess CT’s diagnostic utility. Data from prior imaging modalities (CT, radiography, MRI, ultrasound), microbiological assays (blood cultures, bronchoalveolar lavage, urinalysis), and enzymatic profiles (transaminases, pancreatic enzymes) were included. The predictive value of clinical and laboratory parameters was evaluated via correlation analysis. **Results:** A total of 112 patients were evaluated, with 99 exhibiting 147 inflammatory foci (92 thoracic, 55 abdominal). Definitive diagnoses were made in 58.5% of cases, while 41.5% remained classified as possible. Prior diagnostic procedures identified inflammatory origins in 57.1% of cases. Fewer CT-detected foci were observed in patients with bronchial asthma or type 2 diabetes mellitus (*p* = 0.049 and *p* = 0.006). **Conclusions:** CT imaging plays a central role in identifying infectious foci in ICU patients with unexplained syndromes, particularly in the thoracic region. CT scanning is recommended for sepsis management when other diagnostic evidence is lacking. Conditions such as bronchial asthma or diabetes mellitus may prompt earlier suspicion of infectious foci due to elevated inflammatory markers.

## 1. Introduction

In the intensive care setting, computed tomography (CT) is invaluable due to its rapid and reliable imaging capabilities, particularly for patients who may struggle to cooperate with other imaging procedures. CT is the preferred modality for evaluating fever/inflammation of unknown origin, which is common in critically ill patients [1]. Its superiority over conventional radiography and ultrasound is well documented, with studies consistently demonstrating CT’s enhanced diagnostic precision [2,3,4]. This precision is crucial for the early and accurate identification of infectious processes, directly impacting patient management and outcomes in the critical care environment [5,6].

Performing CT scans on intensive care patients is logistically challenging due to the significant support these patients often require, such as mechanical ventilation, vasopressors, continuous sedation, or even systemic support like extracorporeal membrane oxygenation (ECMO). These complexities demand meticulous planning to ensure both patient safety and diagnostic accuracy during transport and the execution of CT procedures [7,8,9,10,11].

The increasing demand for computed tomography (CT) scans in vulnerable ICU populations requires a careful evaluation of their diagnostic efficacy relative to potential risks. These include radiation exposure, intolerance reactions to the administration of iodine-based contrast agents, and the transport of the patient from the ward to the radiology department, as outlined in various studies [7,8,12,13]. Rigorous assessment of its diagnostic yield is crucial to ensure that the use of CT truly enhances patient outcomes [14].

This study aims to critically evaluate the diagnostic accuracy of CT in identifying infectious foci in intensive care settings. Additionally, it explores the correlation between clinical observations, laboratory parameters, and CT findings to assess their potential utility in predicting the presence of these infectious sites.

## 2. Materials and Methods

### 2.1. Patient Cohort

This prospective study enrolled ICU patients between July 2016 and October 2018 who underwent combined chest and abdominal CT scans to investigate potential unidentified inflammatory foci. Patient selection followed a structured three-step inclusion process to ensure clinical relevance and homogeneity.

First, only patients who were receiving intensive care treatment at the time of imaging were considered eligible. To maintain cohort consistency, patients were exclusively recruited from designated medical and surgical ICUs, while CT examinations ordered from non-ICU wards or outpatient settings were excluded.

Second, the primary indication for CT imaging was the detection of an unknown infectious focus. The referral indications were systematically analyzed, and only cases where the leading clinical question explicitly stated a search for an unknown inflammatory focus were included. Relevant indications comprised terms such as “infection source search”, “elevated inflammatory markers, focus search”, or “unclear systemic inflammation, localization?”. Examinations primarily conducted for alternative reasons, such as suspected free air, ileus clarification, or foreign body placement assessment, were excluded unless infection detection remained the predominant objective. Furthermore, only scans covering both the thorax and the abdomen were included, as partial scans were deemed insufficient for a comprehensive infection assessment.

**Figure 1:**
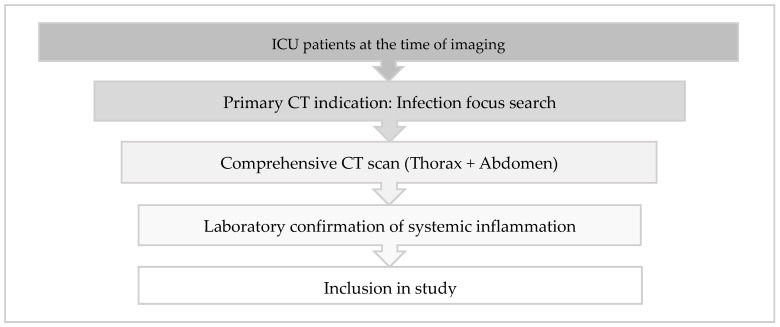
Inclusion criteria for the enrollment of CT examinations in the study.

Third, laboratory confirmation of systemic inflammation was required. At least one inflammatory marker had to exceed standard thresholds, including C-reactive protein (CRP) > 5 mg/L, procalcitonin (PCT) > 0.5 µg/L, white blood cell count (WBC) > 10,000/µL, or body temperature > 37.5 °C. Only examinations fulfilling all three criteria were included in the study (refer to Figure 1). Ethical approval was granted by the local ethics committee, including a waiver for patient consent (application number 002/15, approval date 27 January 2016).

### 2.2. Image Acquisition

CT imaging was performed on multislice spiral CTs, primarily utilizing a 256-slice MDCT (Philips iCT Brilliance 256, Amsterdam, the Netherlands). This was complemented by a subset of scans performed with 16-slice and 124-slice MDCT units (Philips Brilliance 16 and Philips IQon Spectral CT, respectively) (see Table 1).

Oral and/or intravenous contrast agents were utilized in the majority of CT scans (*n* = 109). Intravenous contrast was administered using a weight-adjusted dosage of iodinated contrast agent (Accupaque 300 mg/mL, GE Healthcare Buchler GmbH & Co. KG, Solingen, Germany). The contrast agent was delivered in a monophasic bolus at a flow rate of 3 mL/s, followed by a 40 mL saline flush. Dosage was tailored based on the patient’s body mass index, ranging from 60 to 90 mL. The post-injection delay before scanning was standardized at 60 s. For bowel opacification, oral contrast (Gastrografin^®^, Bayer Vital GmbH, Leverkusen, Germany) was administered prior to the scan.

### 2.3. Diagnostic Imaging Analysis

Interpretations of the radiological images were conducted by at least two consultant radiologists, each with a minimum of five years of experience, utilizing the DeepUnity PACS system (Dedalus, Bonn, Germany). The findings were categorized into thoracic and abdominal regions, with organ localization noted for each identified focus. Individual foci were distinctly identified based on their specific organ localization or demarcation from the morphologically normal surrounding tissue. Furthermore, focal findings were classified as either definite or possible, depending on their morphological characteristics.

### 2.4. Novel Focus Identification

Inflammatory foci were classified as new if they were not previously identified in any recent radiological examinations, including CT, X-ray, ultrasound, or MRI, conducted during the same ICU stay. Additionally, the absence of any prior indication from microbiological evaluations, such as bronchoalveolar lavage, blood cultures, and urinalysis, or from organ-specific chemical laboratory tests (e.g., lipase, α-amylase, alanine aminotransferase, aspartate aminotransferase), also qualified foci as new. For radiological pre-diagnostics, the analysis was limited to the most current study, except in the case of conventional radiographs, where the last three examinations conducted were considered. The focal findings were then classified in the radiologists’ reports as either definite or possible based on morphologic characteristics.

### 2.5. Clinical and Laboratory Chemical Parameters

#### 2.5.1. Vital and Respiratory Parameters

Vital signs and ventilatory parameters were recorded either manually on trace documentation or digitally through the Patient Data Management System (PDMS), with the method used depending on the prevailing documentation practices. Handwritten traces were digitized and archived within the Hospital Information System (HIS), accessible as scanned copies. In both scenarios, these parameters were documented consistently at 10-minute intervals. Among the 112 patients studied, 13 had their data recorded manually, while the remaining 99 were monitored via the PDMS. Data extraction focused on the 60 min period immediately preceding the CT scan to minimize confounding variables such as physiological changes during transport from the ICU to the radiology department. For those disconnected from the PDMS for over 60 min prior to their CT scan (*n* = 13), the last recorded data before disconnection were used. The mean duration between the disconnection and the CT scan for these patients was 94 min.

#### 2.5.2. Medication

Information on prior medications administered to patients was obtained from either digital records within the Patient Data Management System (PDMS) or analog documentation. The analysis was restricted to drug classes commonly used in critical care and/or those with potential implications for inflammatory manifestations: catecholamines (e.g., norepinephrine, dobutamine), sedatives (e.g., propofol, midazolam), antibiotics (e.g., tazobactam, meropenem), and immunomodulators (e.g., glucocorticoids, biologics, chemotherapy).

#### 2.5.3. Blood Parameters, Microbiology, Body Temperature, and Medical History

Comprehensive data, including laboratory chemistry, microbiology, body temperature, and medical history, were sourced from Hospital Information System (HIS) records.

Key inflammatory markers such as C-reactive protein (CRP), procalcitonin (PCT), and white blood cell (WBC) count were evaluated using values from the last blood sample taken prior to the CT scan, which was on average 9 h and 18 min before imaging. Arterial blood gas (ABG) analyses, including measurements of pH, pO_2_, pCO_2_, base excess, O_2_ saturation, hemoglobin (Hb), potassium, and lactate levels, were based on the most recent specimens collected, on average 2 h and 33 min before CT. Body temperature measurements were conducted using probes in urinary catheters. The presence of pre-existing medical conditions was verified through both internal and external medical correspondence and consultation reports.

The study also considered the most recent results of selected microbiological and enzymatic tests indicative of focal inflammation, such as blood cultures, bronchoalveolar lavage (BAL), urinalysis, pancreatic enzymes, and transaminases, with the inclusion criterion being that these tests must have been conducted within the last 10 days. Microbiological and enzymatic assessments were selectively analyzed for patients exhibiting corresponding inflammatory foci identified on CT. For example, results from a prior BAL were included only if a pulmonary focus was indicated on the CT scan. Blood cultures were systematically considered for all patients with an identified focus in the CT (*n* = 99) to evaluate potential sources of bacterial dissemination into the bloodstream. However, blood cultures were actually obtained prior to CT in only 52 of these 99 patients.

### 2.6. Statistical Analysis

Statistical analyses were conducted using SPSS version 25 (IBM, Armonk, NY, USA) and R version 3.5.2 (The R Foundation for Statistical Computing, Vienna, Austria). The analysis included comparative assessments of nominal variables such as sex and contrast protocol, which were examined using cross-tabulations and χ^2^ (chi-square) tests. Metric variables, including age and vital signs, were analyzed using t-tests. The threshold for statistical significance was established at *p* < 0.05.

## 3. Results

### 3.1. Patient Demographics and CT Scan Characteristics

The study evaluated 112 critical care patients who underwent comprehensive CT imaging of the chest and abdomen. The demographic breakdown included 34.8% female participants, with ages ranging from 19 to 88 years (mean age 64.8 ± 13.8 years) (refer to Table 2). Notable chronic conditions were prevalent among the cohort, with congestive heart failure present in 28.6% (*n* = 32) of the subjects, diabetes mellitus in 26.8% (*n* = 30), chronic obstructive pulmonary disease (COPD) in 18.8% (*n* = 21), and bronchial asthma in 7.1% (*n* = 8).

Patients were recruited from six specialized ICU departments: Of the 112 cases included, 30.4% (*n* = 34) were being treated in the general surgery ICU during acquisition of CT, 26 in the anesthesia ICU (23.2%), 19 in the cardiac surgical ICU (17.0%), 15 in the medical ICU (13.4%), 11 in the hemato-oncology ICU (9.8%), and 7 in the neurosurgical ICU (6.2%). Thus, a cohort of 60 patients (53.6%) was being treated in surgical ICUs (general surgery, cardiac surgery, neurosurgery), comprising postoperative subjects after relevant surgical, neurosurgical, and cardiac surgical procedures. In total, 52 patients (46.4%) were recruited from ICUs of non-surgical specialties (medical, hemato-oncology, anesthesiology), primarily comprising patients with internal medicine-related conditions, such as respiratory compromise, systemic inflammatory response syndrome (SIRS), or those with neutropenic fever.

A total of 93 patients (83.0%) were receiving catecholamine therapy, 75 patients (67.0%) were undergoing intravenous sedation, 102 patients (91.1%) were on antibiotic therapy, and 30 patients (26.8%) were receiving immunomodulation therapy.

Contrast-enhanced scans were acquired in 109 patients (97.3%). Combined intravenous and oral contrast was used in 83.9% (*n* = 94) of the scans. Exclusively intravenous contrast was used in 7.1% (*n* = 8) of the cases, and solely oral contrast in 6.3% (*n* = 7). A small subset, 2.7% (*n* = 3), underwent scanning without any contrast medium, primarily due to contrast intolerance, renal insufficiency (GFR < 30 mL/min), or pre-existing thyroid conditions. Non-use of oral contrast was generally due to intolerance, nausea, vomiting, or an increased risk of aspiration, necessitating native CT scans in these instances.

### 3.2. Focus Findings

#### 3.2.1. Frequency and Distribution of Focal Findings

In the cohort of 112 critical care patients, a total of 147 inflammatory foci were identified in 99 patients. The distribution of these foci across the CT scans was as follows: 53.6% of the scans revealed a single focus, 26.8% identified double foci, and 8.0% showed triple foci. Notably, 11.6% of the scans did not identify any inflammatory causes. Consequently, 88.4% of the CT scans detected at least one inflammatory lesion, with a mean of 1.48 lesions per patient.

#### 3.2.2. Radiological Quality of Focus Findings

Out of 147 identified inflammatory foci, radiologic evaluations classified 86 (58.5%) as definitive, indicating a high degree of diagnostic certainty. The remaining 61 foci (41.5%) were categorized as possible, reflecting less certainty in their inflammatory nature. Detailed analysis of the CT scans revealed that 64 scans (57.1%) identified a single definitive focus, while 11 scans (9.8%) showed exactly two definitive foci. In total, 75 of the 112 CT scans (67.0%) detected at least one definitive inflammatory focus.

Conversely, 37 scans (33%) showed either no discernible foci (*n* = 13) or only possible foci (*n* = 24).

#### 3.2.3. Localization of Focal Findings

**Figure 2:**
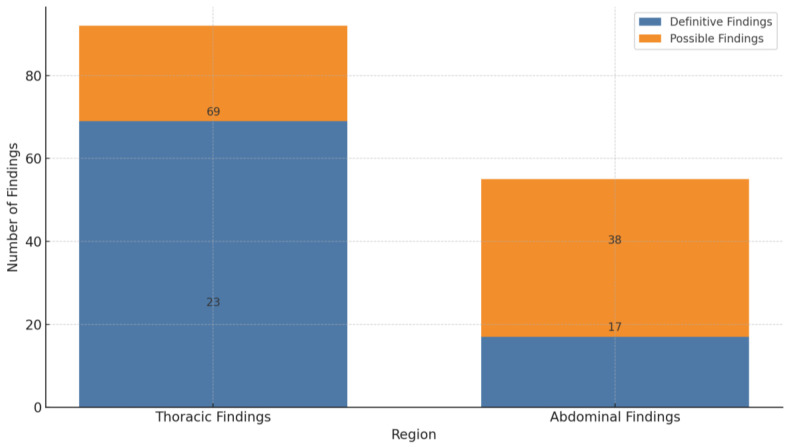
Frequency and certainty of thoracic and abdominal findings on CT.

The study identified 92 focus findings (62.6%) within the thoracic region and 55 focus findings (37.4%) in the abdominal region. A detailed analysis of these findings indicated a significant dichotomy in diagnostic certainty. In the thoracic area, 69 foci (75%) were definitively characterized as inflammatory, while 23 (25%) were classified as possible. In contrast, abdominal foci demonstrated a higher degree of diagnostic uncertainty, with only 17 (30.9%) categorized as definite and the majority, 38 (69.1%), as possible (refer to Figure 2).

**Figure 3:**
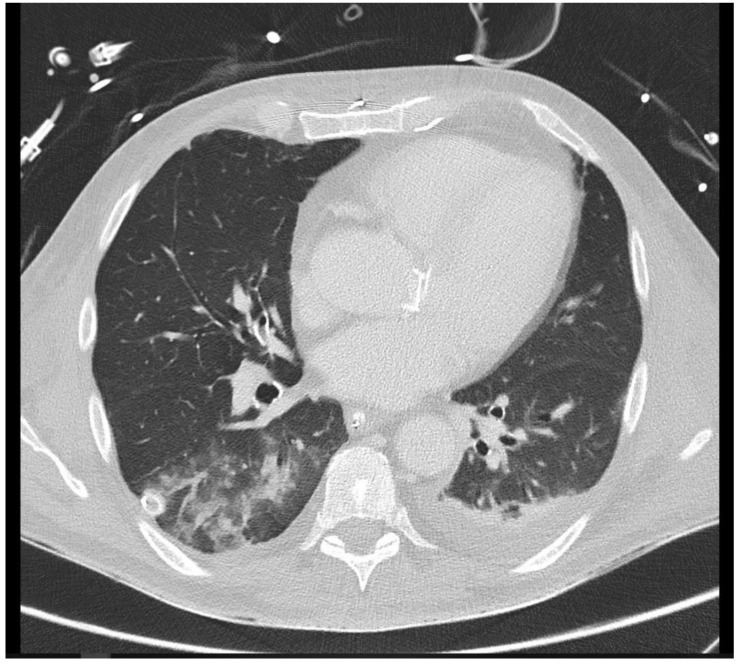
A 26-year-old intensive care patient with elevated inflammatory markers. Typical morphologic signs of pneumonia in the right lower lobe are evident, including ground-glass opacity (GGO), bronchial wall thickening, and pleural effusion.

The distribution of thoracic foci was concentrated primarily in three areas: the lungs, mediastinum, and sternum. Of the thoracic findings, 90 foci were present in the lungs, representing 97.8% of the total, while the mediastinum and sternum each contained one focus (refer to Table 3). Pulmonary foci demonstrated high diagnostic conclusiveness, with 75.6% definitively diagnosed. This highlights the effectiveness of CT imaging in accurately detecting and characterizing lung-associated inflammatory processes, as exemplified in Figure 3.

**Figure 4:**
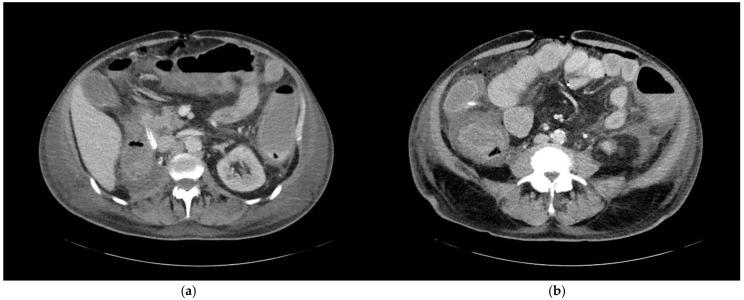
A 68-year-old patient with an unknown focus of origin. CT morphologic signs of colitis include (**a**) circular colonic wall thickening and pericolic fat stranding, and (**b**) decreased mural contrast enhancement indicating intestinal wall edema and adjacent ascites.

Abdominal foci were distributed across 16 distinct anatomical locations, with the colon identified as the most common site, harboring 14 foci (refer to Table 4 and Figure 4). Morphological analysis from imaging studies revealed that only 42.9% of these colonic findings were categorized as definite, reflecting some diagnostic ambiguity in distinguishing inflammatory conditions within this region. Following the colon, the pancreas and the small intestine were the next most frequent sites of inflammation, with 12 and 6 foci, respectively. Additionally, a single focus in the kidney was definitively identified, highlighting both the diversity and the varying levels of diagnostic clarity associated with abdominal foci.

### 3.3. Infectious Foci in the Context of Preliminary Diagnostics

**Figure 5:**
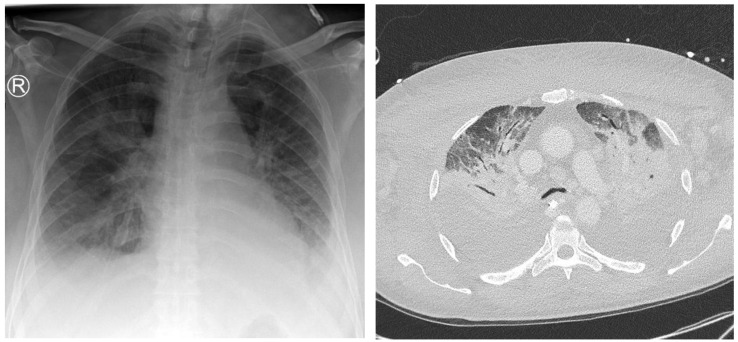
A 75-year-old patient with a focus of unknown origin and uncertain signs of pneumonia on preliminary radiography. Left: Radiograph showing bilateral opacities in the central and inferior fields. Right: CT scan showing extensive bilateral pulmonary infiltrate and pleural effusion.

In the evaluation of the 147 infectious foci identified, 80 (54.4%) had been recognized in at least one previous radiologic examination. A substantial disparity was observed between the anatomical locations: thoracic foci had a higher rate of prior detection, with 68 out of 92 (73.9%) previously reported, whereas only 12 out of 55 abdominal foci (21.8%) had been noted in earlier studies. Of the foci revisited, 41 (27.9% of the total) were confirmed with certainty in at least one previous imaging modality, encompassing 34 thoracic and 7 abdominal foci. In total, 81.3% of these previously diagnosed lesions were identified from the last three preliminary radiographs, yet only 36.9% of these cases resulted in a definitive initial diagnosis (refer to Figure 5).

Computed tomography (CT) proved essential, accurately identifying 55% of all previously diagnosed focal lesions and providing a definite preliminary diagnosis in 72.7% of the cases, affirming its status as the most reliable diagnostic modality. In comparison, despite the limited number of cases (*n* = 2), preliminary ultrasound examinations delivered definite radiological findings in 50% of the instances. However, two preliminary MRI scans failed to indicate any inflammatory process that was later diagnosed via other modalities.

Microbiologic analysis of 52 blood cultures identified pathogens in 13 cases, isolating various pathogens such as *Candida albicans*, *Enterococcus faecalis*, *Enterococcus faecium*, *Klebsiella oxytoca*, *Klebsiella pneumoniae*, *Staphylococcus aureus*, *Staphylococcus epidermidis*, and *Streptococcus intermedius*. However, these microbiologic findings did not aid in pinpointing specific infectious foci.

Further diagnostic tools included bronchoalveolar lavage (BAL) in three cases, pancreatic enzymes in ten cases, and transaminases in one case, which collectively suggested an inflammatory focus in eight cases. Of the 80 foci previously identified via radiology, four were subsequently validated by earlier pancreatic enzyme tests (*n* = 2) or BAL (*n* = 2). Additionally, four instances of suspected pancreatitis, indicated by elevated pancreatic enzymes, lacked corresponding infectious foci in prior imaging assessments. It is important to note that no urine culture results from within the last 10 days were available for patients who underwent CT scanning to identify infectious foci.

In conclusion, 84 of the 147 infectious foci identified (57.1%) were detected in at least one previous imaging modality, bronchoalveolar lavage (BAL), or pancreatic enzyme analysis, indicating a significant rate of pre-identification. On the other hand, 63 foci (42.9%) were newly discovered findings, not identified in any prior diagnostic evaluations, affecting 46 patients (41.1%).

### 3.4. Prognostic Value of Clinical and Laboratory Chemical Parameters

#### 3.4.1. Age, Sex, Pre-Existing Conditions, and Medication

The study found no significant age difference between patients with and without inflammatory findings, with mean ages of 64.2 and 68.8 years, respectively (*p* = 0.266). The gender distribution among the 99 patients with identified foci was 66.7% male and 33.3% female, which reflects the overall patient cohort distribution (90.4% of males and 84.6% of females had at least one focal finding, *p* = 0.37).

Regarding pre-existing conditions among those with focal findings, the prevalence was as follows: 29.3% had congestive heart failure, 5.1% had bronchial asthma, 17.2% had chronic obstructive pulmonary disease (COPD), and 22.2% had diabetes mellitus. For the 13 patients without focal findings, the corresponding figures were 23.1% for heart failure, 23.1% for asthma, 30.8% for COPD, and 61.5% for diabetes mellitus. Interestingly, while the presence of congestive heart failure or COPD showed no significant correlation with the incidence of inflammatory foci, asthma (*p* = 0.049) and diabetes mellitus (*p* = 0.006) were associated with a significantly lower frequency of inflammatory foci identified on the CT scans.

Among 93 patients on catecholamine therapy, at least one inflammatory focus was detected on CT in 82 patients (88.2%), compared to 13 of 15 patients (86.7%) without catecholamine support (*p* = 1.000). Similar distributions were found for intravenous sedation (66/75, 88.0%) and antibiotic therapy (89/102, 87.3%), with comparable rates in patients without these therapies (87.9% and 100%, respectively; *p* = 1.000).

Immunomodulatory therapy was associated with inflammatory foci in 26 of 30 patients (86.7%), compared to 69 of 78 (88.5%) patients without (*p* = 0.752).

No statistically significant association was found between any of the therapies and inflammatory focus detection on CT.

#### 3.4.2. Impact of Contrast Administration on Detection of Inflammatory Foci

This study examined the relationship between the administration of contrast media and the detection of inflammatory foci during CT scans. Statistical analysis revealed no significant association between contrast use and the identification of inflammatory foci (*p* = 0.429). Despite the predominant use of both oral and intravenous contrast in 84.8% of scans, these contrast-enhanced scans detected at least one inflammatory focus in 89.4% of cases.

#### 3.4.3. Vital Signs and Shock Index

The analysis aimed to determine whether vital signs, including heart rate and blood pressure, could serve as predictors for the presence of inflammatory lesions. The results showed no significant differences in these vital signs between patients with and without focal findings (see Table 5), indicating that such physiological parameters are not reliable predictors of inflammation in this clinical setting (*p* > 0.05).

Furthermore, the shock index, a derived measure used to indicate hemodynamic shock (Allgöwer and Buri, 1967 [15]), was evaluated for its diagnostic utility. The results showed no significant variation between the patient groups (*p* = 0.575).

#### 3.4.4. Ventilation Parameters

The study evaluated whether mechanical ventilation status influenced the detection of inflammatory foci. Among the mechanically ventilated patients (*n* = 90), 90% were found to have at least one inflammatory focus, in contrast to 76.5% of the patients who were breathing spontaneously. However, this difference did not reach statistical significance (*p* = 0.216), suggesting that mechanical ventilation status alone does not predict the presence of inflammatory lesions. Furthermore, no significant correlation was observed between the type of mechanical ventilation utilized and the presence of inflammatory foci (*p* = 0.143). Analysis of specific ventilatory parameters such as peak airway pressure, mean airway pressure, and positive end-expiratory pressure (PEEP) also revealed no significant differences between patients with and without foci (see Table 5).

#### 3.4.5. Laboratory Chemical Parameters

The study analyzed a range of laboratory chemical parameters from arterial blood gas (ABG) tests, including pH, pCO^2^, base excess, pO^2^, O^2^ saturation, hemoglobin, potassium, and lactate levels, as documented in Table 6. Statistical analysis revealed no significant differences between patients with and without inflammatory foci, indicating that these parameters did not differ markedly across groups.

Furthermore, common inflammatory markers such as leukocyte count, procalcitonin (PCT), C-reactive protein (CRP), and body temperature were examined. Like the ABG results, these markers showed negligible differences between the two groups.

## 4. Discussion

The primary aim of this study was to assess the diagnostic efficacy of computed tomography (CT) for identifying infectious foci in ICU patients who present with ambiguous inflammatory conditions. The results proved a high detection capacity, with 88.4% of CT scans identifying at least one inflammatory focus and 67% of scans delineating morphologically distinct foci. Such diagnostic accuracy is critical, emphasizing the indispensable role of CT in the clinical evaluation of sepsis, especially when the origin of the infection is unclear.

This significant diagnostic capability of CT supports its continued use as a primary tool in the investigative protocol for sepsis management in critical care settings [16,17,18]. The ability to detect and characterize infectious foci effectively aids in the prompt and accurate treatment of sepsis, potentially improving patient outcomes in these high-risk scenarios. Moreover, the findings advocate the integration of CT imaging alongside other diagnostic approaches to enhance the overall diagnostic strategy and patient care in intensive care units.

### 4.1. Comparative Diagnostic Yield

This study showed a high success rate in identifying inflammatory foci compared to the existing literature, indicating a substantial diagnostic yield of CT scans in ICU settings. Barkhausen et al. (1999) and Velmahos et al. (1999) reported lower identification rates, underscoring the variability in diagnostic outcomes that can arise from different patient populations and study methodologies [10,17]. Conversely, our findings are in line with more recent studies by Pohlan et al. (2024), Pohlan et al. (2022), Schleder et al. (2017), and Pandharipande et al. (2016), which reported significant alterations in diagnosis following CT evaluations in patients presenting with abdominal and thoracic symptoms [18,19,20,21].

Notably, the prevalence of pneumonia as the primary inflammatory focus in our study and that of Schleder et al. emphasizes the critical role of the lung as a primary site of infection in ICU patients. This needs vigilant monitoring and thorough assessment. The notably high detection rate in our study may be attributed to the exclusive focus on ICU patients and the comprehensive approach of including both thoracic and abdominal scans. This methodological focus likely enhanced the proportion of CT scans that successfully identified focal findings, as compared to other studies that encompassed more restricted examination scopes or mixed patient populations.

Our study highlights a distinct difference in the distribution and diagnostic certainty of inflammatory foci between thoracic and abdominal regions on CT imaging. Specifically, thoracic findings had a higher proportion of definitive diagnoses (75%), while abdominal findings exhibited a higher incidence of possible diagnoses (69.1%). The superior diagnostic certainty in the thoracic region can be attributed to the clear visualization of pulmonary structures and common infection indicators, such as ground-glass opacities and pleural effusions. These features facilitate a more confident interpretation by radiologists, resulting in a higher rate of definitive diagnoses. Conversely, the abdominal region presents greater diagnostic challenges due to its anatomical complexity and the varied nature of potential pathologies. Conditions like colitis and pancreatitis often have less distinct morphological features, complicating their interpretation and leading to a higher proportion of possible findings. This disparity underscores the need for supplementary diagnostic methods in abdominal cases. Integrating advanced imaging techniques and correlating findings with clinical and laboratory data can enhance diagnostic accuracy for abdominal infections.

In summary, CT imaging proves highly effective for thoracic infections in ICU patients, given its high diagnostic certainty. However, the diagnosis of abdominal infections requires a more comprehensive approach due to the higher incidence of ambiguous findings. Future efforts should aim to refine imaging techniques and incorporate multimodal diagnostics to improve the detection and characterization of abdominal infectious foci.

### 4.2. Methodological Considerations

Our study refines the radiologic assessment of infectious foci by explicitly distinguishing between definite and possible CT findings, enhancing the understanding of the radiologic characterization of infectious foci. A similar approach was previously applied by Pohlan et al. (2022), who categorized 37.5% of foci as certain, 18.9% as likely, and 15.9% as possible [19]. In contrast, our study classified 58.5% as definitive and 41.5% as possible, without an intermediate “likely” category. This may explain the higher proportion of definitive diagnoses in our cohort, as the absence of a “likely” category in our classification system may have led to a clearer delineation between definite and possible findings. Also, differences may stem from differences in patient populations, imaging protocols, or interpretation criteria.

Moreover, the fact that over half of the identified foci had been previously detected or suspected through other imaging modalities underscores the incremental value of CT scans. It highlights the role of CT in confirming or clarifying findings that are initially uncertain, thereby contributing significantly to the identification of suspected infectious processes in critical care settings.

### 4.3. Clinical Implications

The utility of CT scans extends beyond initial imaging evaluations, particularly when previous assessments have indicated potential infectious processes. This utility is critical in scenarios involving clinical-laboratory discrepancies, the reappearance of previously identified foci, or the emergence of new clinical signs that necessitate a reevaluation of existing diagnoses or the identification of additional infectious foci. While numerous radiographic signs of pneumonia are often noted, definitive diagnosis remains scarce, underscoring the value of CT in follow-up assessments and further characterization of foci. This includes detailed analyses such as typing and quantification of pulmonary infiltrates, detection of focal atelectasis, bronchial obstruction, and identifying associated clinical symptoms.

Moreover, our study identifies a statistically significant correlation between the presence of the chronic inflammatory conditions of bronchial asthma and diabetes mellitus, and lower detection rates of inflammatory foci on CT scans. These conditions are known to contribute to systemic inflammatory responses, potentially elevating systemic inflammatory markers and complicating the interpretation of these parameters in clinical diagnostics [22,23,24,25]. The increased inflammatory markers in patients with bronchial asthma and diabetes might reflect an underlying chronic inflammatory state rather than an acute infectious process. Additionally, diabetes mellitus increases the risk of urinary tract infections, often detected through microbiological analysis rather than CT imaging, highlighting the challenges in diagnosing infectious foci in such patients [26,27,28].

In our study, the examination of inflammatory markers, arterial blood gas (ABG) parameters, and other clinical indicators revealed only minor differences between patients with and without identified focal findings. Notably, those with focal findings tended to have slightly elevated levels of lactate, C-reactive protein (CRP), and procalcitonin, alongside a slightly lower mean age, increased heart rate, and elevated systolic blood pressure. Despite these differences, these variations did not significantly correlate with the detection of inflammatory foci on CT scans. This lack of significant correlation emphasizes that, while useful for general clinical assessments, these laboratory parameters do not reliably predict the presence or localization of inflammatory foci.

Furthermore, although preliminary microbiological tests, pancreatic enzymes, and transaminases provided some diagnostic insights in a limited subset of cases (8 out of 147), CT imaging proved to be the most effective tool for identifying inflammatory foci. This underscores the superior diagnostic utility of CT imaging in this clinical context, outperforming other methods in terms of specificity and reliability for detecting and localizing inflammatory processes.

### 4.4. Limitations

While our study presents compelling evidence of the diagnostic capabilities of CT imaging for identifying infectious foci in ICU patients, it is not without limitations. A primary constraint of this study is the relatively small sample size, which precludes the use of extensive multivariate analyses. While our statistical approach allows for meaningful comparisons, it does not account for potential confounders in a multivariate framework.

Another major limitation lies in the scope of the pretest diagnostics considered. Currently, the study primarily relies on a select set of microbiologic tests and standard imaging modalities. Expanding these to include a wider array of microbiologic investigations could provide a more comprehensive understanding of the infectious processes at play.

Additionally, integrating a broader spectrum of diagnostic techniques, such as advanced molecular diagnostics, immune profiling, or newer imaging technologies, could significantly enhance our ability to detect, characterize, and monitor infectious foci. Such advancements could lead to improved diagnostic accuracy and better tailored therapeutic strategies.

The variability in the timing of laboratory tests relative to CT scans represents a methodological limitation. As laboratory values were obtained as part of routine clinical care, the interval between blood sampling and imaging depended on the urgency of the clinical situation and the time of CT indication. While this reflects real-world clinical decision-making, it introduces heterogeneity in the temporal relationship between laboratory parameters and imaging findings.

Furthermore, our characterization of the study population was limited to key demographic factors and selected comorbidities. The lack of standardized metrics, such as the Charlson comorbidity index (CCI), complicates the comparability of study populations across different investigations. Similarly, for assessing the potential predictive value of clinical and laboratory parameters, established scoring systems—such as severity indices like the Sequential Organ Failure Assessment (SOFA) score—could provide a more comprehensive evaluation. Future research should explore the integration of such standardized measures to enhance the generalizability and clinical applicability of the findings.

## 5. Conclusions

This study underscores the vital role of CT imaging within the critical care setting, particularly for identifying infectious foci where the source of infection is not immediately evident. The diagnostic capabilities of CT not only demonstrate its essential value but also highlight its critical role in the comprehensive clinical evaluation of septic patients. These capabilities facilitate improved diagnostic accuracy and aid in the formulation of targeted treatment strategies.

This study highlights the critical role of CT imaging in diagnosing infectious foci in ICU patients, with thoracic scans showing higher diagnostic certainty due to clearer visualization of pulmonary structures. Abdominal scans, however, present more diagnostic challenges and higher ambiguity, necessitating supplementary diagnostic methods. While CT is highly effective for thoracic infections, a comprehensive, multimodal approach is essential for accurately diagnosing abdominal infections. Future research should focus on refining imaging techniques and integrating clinical and laboratory data to enhance diagnostic accuracy.

Looking forward, it is imperative to broaden the diagnostic framework used in such studies by integrating a more diverse array of preliminary tests. This enhancement would allow for a deeper understanding of the relationships between clinical presentations, underlying chronic conditions, and the detectability of infectious foci. Moreover, further research investigating the direct impact of CT scans on therapeutic outcomes and overall patient prognosis in cases of systemic inflammatory response syndrome (SIRS), sepsis, and infections of unknown origin could yield invaluable insights into the broader utility and efficacy of CT imaging in these complex scenarios.

## Figures and Tables

**Table 1:** Examination parameters of all multislice spiral CT used.

CT Type	Philips iCT Brilliance 256	Philips Brilliance 16	Philips IQon Spectral CT
Tube voltage (kV)	120–140 kV	120 kV	120–140 kV
Pitch	0.76	0.798	0.798
Slices	256	16	124
Collimation (mm)	0.625 mm	1.5 mm	0.625 mm
Reconstruction slice thickness (mm)	2	2	2

**Table 2:** Patient demographics and premedication.

Patient Demographics	Total (n = 112)	Surgical ICU (n = 60)	Non-Surgical ICU (n = 52)
Age (mean ± SD)	64.8 ± 13.8 years	55.6 ± 21.9 years	68.3 ± 13.0 years
Sex	73 male/39 female	42 male/18 female	31 male/21 female
Congestive heart failure	32 (28.6%)	18 (30%)	14 (26.9%)
Diabetes mellitus	30 (26.8%)	12 (20%)	18 (34.6%)
COPD	21 (18.8%)	8 (13.3%)	13 (25%)
Bronchial asthma	8 (7.1%)	3 (5%)	5 (9.6%)
Catecholamine support	93 (83.0%)	53 (88.3%)	40 (77.0%)
Sedatives	75 (67.0%)	43 (71.7%)	32 (61.5%)
Antibiotic use	102 (91.1%)	57 (95%)	45 (86.5%)
Immunomodulation	30 (26.8%)	20 (35.1%)	10 (19.2%)

**Table 3:** Thoracic focus findings.

Thorax Examination Area	Number of Foci	Definite Foci	Possible Foci	Proportion of Total Thoracic Focus Findings (*n* = 92)	Proportion of Total Number of Focus Findings (*n* = 147)
Lung	90	68	22	97.8%	61.2%
Mediastinum	1	0	1	1.1%	0.7%
Sternum	1	1	0	1.1%	0.7%
Sum	92	69	23	100%	62.6%

**Table 4:** Abdominal focus findings.

Abdomen Examination Area	*n* Foci	Thereof Definite	Thereof Possible	Proportion of Total Abdominal Focus Findings (*n* = 55)	Proportion of Total Number of Focus Findings (*n* = 147)
Colon	14	6	8	25.5%	9.5%
Pancreas	12	6	6	21.8%	8.2%
Small intestine	6	2	4	10.9%	4.1%
Gallbladder	5	0	5	9.1%	3.4%
Colon + small intestine (Enterocolitis)	4	2	2	7.3%	2.7%
Intraabdominal abscess	3	0	3	5.5%	1.4%
Stomach	2	0	2	3.6%	1.4%
Abdominal wall	2	0	2	3.6%	1.4%
Duodenum	1	0	1	1.8%	0.7%
Aorta	1	0	1	1.8%	0.7%
Intervertebral disk	1	0	1	1.8%	0.7%
Liver	1	0	1	1.8%	0.7%
Kidney	1	1	0	1.8%	0.7%
Peritoneum	1	0	1	1.8%	0.7%
Prostate	1	0	1	1.8%	0.7%
Sum	55	17	38	100%	37.4%

**Table 5:** Mean values of vital signs and ventilation parameters.

		Vital Signs					Ventilation Parameters			
Focus Finding		Heart Rate	SBP	MAD	DBP	Shock Index	Breathing Rate	Ppeak	Pmean	PEEP
yes	n	95	95	95	95	95	90	80	80	80
	mean value	96.09	121.00	75.92	55.17	0.82	20.18	23.78	17.25	12.30
no	n	13	13	13	13	13	12	9	9	9
	mean value	88.69	118.23	79.23	57.85	0.78	19.83	22.89	17.44	12.03
sum	n	110	110	110	110	110	102	89	89	89
	mean value	95.20	120.67	76.31	55.49	0.82	20.13	23.69	17.27	12.27

**Table 6:** Mean values of arterial blood gas parameters and inflammation markers.

		Arterial Blood Gas (ABG)								Inflammation Markers			
Focus Finding		pH	pCO_2_	Base Excess	pO_2_	O_2_	Hb	Potassium	Lactate	WBC Count	Procalcitonin	CRP	Temperature
yes	n	97	97	97	97	97	97	97	97	97	93	61	97
	mean value	7.38	45.13	0.18	104.50	96.32	8.96	4.53	2.36	17.59	10.51	117.05	37.14
no	n	13	13	13	13	13	13	13	13	13	13	10	11
	mean value	7.39	42.70	0.15	93.25	94.96	9.76	4.57	1.49	19.10	6.72	105.66	37.41
sum	n	110	110	110	110	110	110	110	110	110	106	71	108
	mean value	7.38	44.84	0.17	103.17	96.16	9.05	4.53	2.26	17.77	10.04	115.44	37.17

## Data Availability

The data presented in this study are available on request from the corresponding author.
